# Redox balance is key to explaining full *vs*. partial switching to low-yield metabolism

**DOI:** 10.1186/1752-0509-6-22

**Published:** 2012-03-24

**Authors:** Milan JA van Hoek, Roeland MH Merks

**Affiliations:** 1 Centrum Wiskunde & Informatica, Life Sciences, Science Park 123, 1098 XG Amsterdam, The Netherlands; 2 Netherlands Institute for Systems Biology, Science Park 123, 1098 XG Amsterdam, The Netherlands; 3 Netherlands Consortium for Systems Biology, Amsterdam, The Netherlands; 4 Mathematical Institute, Leiden University, P.O. Box 9512, 2300 RA Leiden, The Netherlands

**Keywords:** Metabolic switching, Genome-scale metabolic model, Flux Balance Analysis with Molecular Crowding, Overflow metabolism, Redox balance, Escherichia coli, Lactococcus lactis, Saccharomyces cerevisiae

## Abstract

**Background:**

Low-yield metabolism is a puzzling phenomenon in many unicellular and multicellular organisms. In abundance of glucose, many cells use a highly wasteful fermentation pathway despite the availability of a high-yield pathway, producing many ATP molecules per glucose, *e.g*., oxidative phosphorylation. Some of these organisms, including the lactic acid bacterium *Lactococcus lactis*, downregulate their high-yield pathway in favor of the low-yield pathway. Other organisms, including *Escherichia coli *do not reduce the flux through the high-yield pathway, employing the low-yield pathway in parallel with a fully active high-yield pathway. For what reasons do some species use the high-yield and low-yield pathways concurrently and what makes others downregulate the high-yield pathway? A classic rationale for metabolic fermentation is overflow metabolism. Because the throughput of metabolic pathways is limited, influx of glucose exceeding the pathway's throughput capacity is thought to be redirected into an alternative, low-yield pathway. This overflow metabolism rationale suggests that cells would only use fermentation once the high-yield pathway runs at maximum rate, but it cannot explain why cells would decrease the flux through the high-yield pathway.

**Results:**

Using flux balance analysis with molecular crowding (FBAwMC), a recent extension to flux balance analysis (FBA) that assumes that the total flux through the metabolic network is limited, we investigate the differences between *Saccharomyces cerevisiae *and *L. lactis *that downregulate the high-yield pathway at increasing glucose concentrations, and *E. coli*, which keeps the high-yield pathway functioning at maximal rate. FBAwMC correctly predicts the metabolic switching mode in these three organisms, suggesting that metabolic network architecture is responsible for differences in metabolic switching mode. Based on our analysis, we expect gradual, "overflow-like" switching behavior in organisms that have an additional energy-yielding pathway that does not consume NADH (*e.g*., acetate production in *E. coli*). Flux decrease through the high-yield pathway is expected in organisms in which the high-yield and low-yield pathways compete for NADH. In support of this analysis, a simplified model of metabolic switching suggests that the extra energy generated during acetate production produces an additional optimal growth mode that smoothens the metabolic switch in *E. coli*.

**Conclusions:**

Maintaining redox balance is key to explaining why some microbes decrease the flux through the high-yield pathway, while other microbes use "overflow-like" low-yield metabolism.

## Background

One of the key steps in energy metabolism is to transfer the energy carried by sugars, including glucose, to the biological "energy currency" adenosine triphosphate (ATP). The number of ATP molecules generated by metabolizing one molecule of glucose—the ATP yield—is one of the most basic measures of an organism's energy efficiency. One would perhaps expect that evolution has selected organisms for the ability to extract energy from their food at optimal efficiency by maximizing ATP yield. Yet surprisingly, many organisms switch between a high-yield pathway, *e.g*., aerobic respiration that yields more than thirty moles of ATP per mole glucose, and a highly inefficient, low-yield fermentation pathway that yields only two or three moles ATP per mole of glucose. This effect is known as the Crabtree-effect in the baker's yeast *Saccharomyces cerevisiae. S. cerevisiae *turns glucose into CO_2 _in aerobic, glucose-limited conditions. But in abundance of glucose, glucose is converted into ethanol [1], even if oxygen levels do not limit aerobic metabolism. Many bacteria also use a high-yield metabolic pathway in glucose-limited conditions and a low-yield pathway in excess of glucose. Examples are *Escherichia coli *[2], *Bacillus subtilis *[3] and lactic acid bacteria, *e.g*., *Lactobacillus plantarum *and *Lactococcus lactis *[4,5]. The effect is also found in multicellular eukaryotes, including human cancer cells, where it is called the Warburg effect [6]. Muscle cells switch to low-yield metabolism during heavy exercise [7], fermenting glucose into lactic acid. Why cells would produce less ATP per glucose molecule than they can is a long-standing question in biology [8-12].

Microbial species show remarkable differences in their metabolic switching strategies. At low glucose concentrations and low growth rates, *E. coli *uses high-yield metabolism, aerobically converting glucose into CO_2 _and water. At higher glucose concentrations and fast growth rates, it redirects part of the glucose influx into a low-yield fermentation pathway, keeping oxidative phosphorylation fully active [2]. *S. cerevisiae *uses high-yield, aerobic respiration at slow growth rates; at fast growth rates it ferments most glucose into ethanol, and downregulates aerobic respiration, keeping aerobic respiration active at a much lower rate. Although *L. lactis *does not have an aerobic respiration pathway, it still performs a metabolic switch. At fast growth rates it makes a full switch to lactic acid fermentation [4], which yields about 50% less ATP than the higher-yield mixed acid fermentation pathway, that produces formate, acetate and ethanol.

A plausible explanation for metabolic switching is "overflow metabolism". It assumes that organisms only switch to low-yield metabolism if the high-yield pathway is operating at maximum rate and cannot process any more molecules [13,14]. The remainder would then spill into the low-yield pathway. This explanation requires the low-yield pathway to operate at a faster rate than the high-yield pathway, which is likely the case [8,15,16]. Thus overflow metabolism plausibly explains concurrent use of high-yield and low-yield pathways, as in *E. coli*. However, a problem with overflow metabolism is that it does not explain why organisms like *S. cerevisiae *or *L. lactis *would partly switch off their high-yield pathways at high growth rates.

Recent studies have suggested that the limited amount of metabolic enzymes fitting inside the cell may be key to low-yield metabolism [12,17,18]. Simply because cells can host only a finite number of metabolic enzymes, they may need to trade off investment into the bulky enzymatic machinery required for low-throughput, high-yield metabolism, or alternatively to invest into many more "lean" glycolytic enzymes producing a high-throughput, low yield metabolism. Thus, according to this view, high glucose uptake rate should correlate with low yield metabolism, and vice versa. Indeed, this is observed in comparative studies of metabolism in yeast species of the *Saccharomyces *clade [19] and in comparative studies of glucose metabolism of various bacterial species [20].

**Figure 1:**
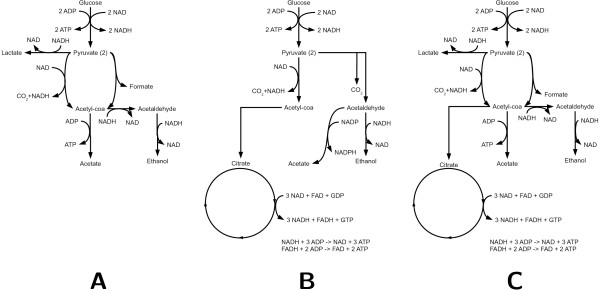
**Simplified reaction scheme for the 3 organisms studied**. A. *L. lactis*; B. *S. cerevisiae*; C. *E. coli*.

If cells need to trade off fast metabolism and high-yield metabolism, then why do we still observe overflow metabolism, as in *E. coli*? We address this question by comparing the optimal metabolic switching strategies of *L. lactis*, *S. cerevisiae*, and *E. coli *as predicted by a genome-scale computational model. These three organisms use different pathways to metabolize glucose. In Figure 1 a simplified reaction scheme of the most important glucose degrading pathways in these three organisms is presented. *E. coli *can use oxidative phosphorylation, lactate fermentation, ethanol fermentation and acetate fermentation. *L. lactis *can use mixed-acid fermentation, producing formate, acetate and ethanol, or lactate fermentation. *S. cerevisiae *can use oxidative phosphorylation, ethanol fermentation and acetate fermentation.

To predict the metabolic switches these three organisms can perform, we make use of a variant of Flux Balance Analysis (FBA), a method that calculates fluxes through metabolic networks given constraints on the network and given an objective function to maximize. By maximizing growth rate, FBA often correctly predicts cellular metabolism, including uptake, excretion and growth rates of cells [2,21]. However, because the glucose uptake rate is fixed in these simulations, growth *yield *(defined as the growth rate divided by the glucose uptake rate) is effectively maximized [16]. Therefore, FBA cannot satisfactorily predict low-yield metabolism.

For this reason, we use an extension of FBA, Flux Balance Analysis with Molecular Crowding (FBAwMC) [17,22]. In contrast to FBA, FBAwMC calculates the optimal flux distribution through a metabolic network under the physiologically-plausible constraint that only a finite number of metabolic enzymes fit into a cell. Because each of the enzymes has a maximum turnover number (*k_cat_*), molecular crowding naturally results in a constraint on the total metabolic flux through the network:

(1) $$\sum c_{i}f_{i}\leq V_{prot},$$

with *f_i _*being the flux through reaction *i*, *V_prot _*the volume fraction of macromolecules devoted to metabolic enzymes and *c_i _*the "crowding coefficient" of reaction *i*. The crowding coefficient of reaction *i *is defined as the volume that needs to be occupied with enzymes to reach unit flux through reaction *i *and is given by $c_{i}\equiv \frac{Mv_{i}}{Vb_{i}},$where *M *is the cell mass, *V *the cell volume, *v_i _*the molar volume of the enzyme catalyzing reaction *i*, and *b_i _*a variable describing the proportionality between enzyme concentration and flux through reaction *i *[22]. Intuitively, the crowding coefficient can be seen as the protein cost of a reaction: enzymes with low crowding coefficients have small molecular volume or catalyse fast reactions. FBAwMC correctly predicts low-yield metabolism: *e.g*., growth curves of *E. coli *[17,22], and the Warburg effect in cancer cells [18]. Therefore, FBAwMC is well suited for our aim: to unravel the metabolic differences between microbes that decrease the flux through the high-yield pathway at high growth rates and those that keep the high-yield pathway always fully active.

Because crowding coefficients for most metabolic enzymes are unknown, previous studies proposed a range of strategies to estimate them. Beg *et al. *[22] fitted an average crowding coefficient 〈c〉 in order to obtain a good match between predicted and measured growth rates. Shlomi *et al. *[23] obtained 15% of crowding coefficients from experimental data and assigned the median of the known crowding coefficient values to the remaining unknown crowding coefficients. Vazquez *et al. *[17] sampled crowding coefficients randomly from a range of physiologically-plausible values obtained from on-line, biochemical databases, and presented averages and variations of the metabolic fluxes predicted for a large random sample of crowding coefficients.

Although the study of an estimated, specific set of crowding coefficients or an average can provide some insight, in reality metabolic networks may operate under an entirely different set of crowding coefficients. Therefore, in the absence of accurate, experimental estimates of crowding coefficients, FBAwMC cannot decide on one real situation. Studying growth yield predictions for large samples of biochemically-plausible sets of crowding coefficients can give more robust insights into the metabolic network than studies with single crowding coefficient estimates, because it reveals what growth yields are most plausible and what are the alternative behaviors of the network.

Our analysis suggests that mechanisms to maintain NAD^+^/NADH ratio are key to the metabolic differences between the two types of metabolic switches. Organisms in which both the high-yield and low-yield pathways reduce NADH may downregulate high-yield metabolism at high growth rates. If organisms have an additional energy-yielding pathway that does not consume NADH (*e.g*., acetate production in *E. coli*), it is optimal to keep both the low-yield and high-yield pathways active at high growth rates.

## Results

### Predicted yield distributions reflect metabolic switching strategy

**Figure 2:**
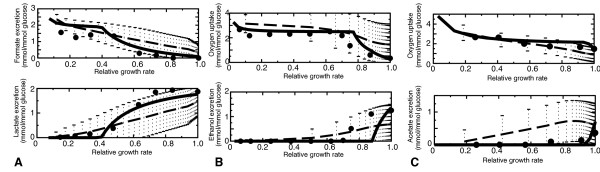
**FBAwMC growth simulations, compared with experimental data (discs)**. The best fitting simulation is indicated with a solid line, the mean and standard deviations with dashed lines. Experimental data are indicated with black dots. We scaled the growth rate of the simulations and the experimental observations to the maximal growth rate. Mean and standard deviations are calculated from all simulations that switch to low-yield metabolism at high growth rates (yield < 0.3 gr dry weight/gr glucose). A. *L. lactis*, data from Thomas *et al. *[4]; B. *S. cerevisiae*, data from Hoek *et al. *[29]; C. *E. coli*, data from Varma and Palsson [2].

Using genome-scale stoichiometric networks of *L. lactis *[24], *E. coli *[25], and *S. cerevisiae *[26] we first confirmed that our implementation of the FBAwMC method reproduces the correct growth curves (Figure 2). Indeed, FBAwMC qualitatively reproduces both the metabolic switch of *S. cerevisiae *and *L. lactis *and the overflow metabolism of *E. coli*. Following Vazquez and coworkers [17], crowding coefficients were chosen at random from a distribution of crowding coefficients based on published molar volumes (Metacyc [27]) and turnover numbers (Brenda [28]). Thus the growth curves that FBAwMC predicts are the average behavior for 1000 randomly sampled sets of crowding coefficients. Figure 2 reports the average and standard deviations of waste product formation or oxygen consumption for the set of simulations resulting in low-yield metabolism (growth yield < 0.3 gr dry weight/gr glucose). This represents 74% of all simulations for *E. coli*, 44% for *L. lactis *and 32% for *S. cerevisiae*. As reflected by the growth curves' large variation, the optimal growth curve that FBAwMC predicts depends strongly on the particular sample of crowding coefficients used. Because a crowding coefficient *c_i _*reflects the enzymatic cost of producing a metabolic flux *f_i_*, we asked if the relation between crowding coefficient selections and the predicted optimal fluxes could yield new biological insight.

**Figure 3:**
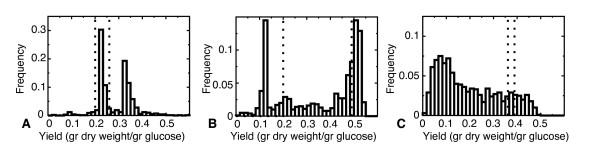
**Distribution of growth yields predicted by the model with 1000 randomly selected sets of crowding coefficients**. A. *L. lactis*; B. *S. cerevisiae*; C. *E. coli*. Dotted vertical lines indicate experimental growth yields for high and low growth rates [2,4,29].

Figure 3 shows the predicted, optimal growth yields at unconstrained glucose influx for a sample of 1000 sets of randomly selected crowding coefficients. Interestingly, computationally obtained growth yields in *L. lactis *and *S. cerevisiae *distribute bimodally, with only few simulations predicting intermediate growth yields. By contrast, the predicted growth yields of *E. coli *are distributed more uniformly. Strikingly, the predicted growth yield distributions for a random sampling of crowding coefficients correlate well with the organisms' switching strategies: *L. lactis *and *S. cerevisiae*, which downregulate their high-yield metabolism, display a bimodal distribution of predicted growth yields, whereas *E. coli*, which has overflow-like metabolism, displays a uniform distribution of predicted growth yields.

**Figure 4:**
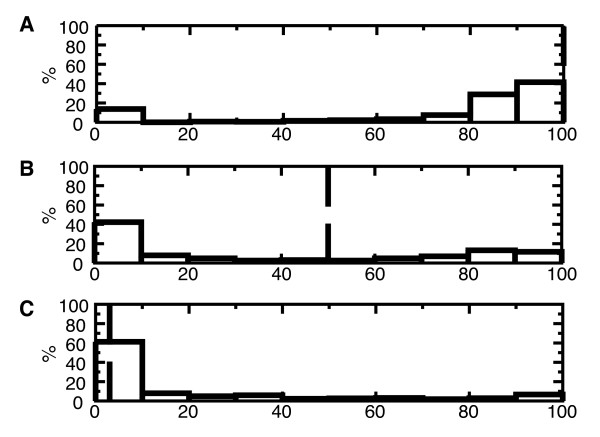
**Flux decrease through the high-yield pathway, relative to the maximum flux through the high-yield pathway**. This is a measure of the decrease in flux through the high-yield pathway during the metabolic switch. As in Figure 2, we only report simulations that resulted in low-yield metabolism, with yield < 0.3 gr dry weight/gr glucose. Dashed lines indicate experimental values. A. *L. lactis*, reported is decrease in formate production rate, data from Thomas *et al. *[4]; B. *S. cerevisiae*, reported is decrease in oxygen uptake rate, data from Hoek *et al. *[29]; C. *E. coli*, reported is decrease in oxygen uptake rate, data from Varma and Palsson [2].

The distribution of growth yields only gives information of the metabolic behavior at maximal growth rates. Next we tested if individual simulations show overflow-like metabolism or not. Figure 4 shows a frequency diagram of the reduction of the flux through the high-yield pathway at maximal growth rate, as a percentage of the maximal flux through the high-yield pathway. For *E. coli *we observe that most sets of crowding coefficients lead to little flux decrease through the high-yield pathway (Figure 4C). However, for *L. lactis*, most sets of crowding coefficients produce a complete halt of the high-yield pathway (Figure 4A). For *S. cerevisiae*, for 40% of crowding coefficients the high-yield pathway is repressed more than two-fold at high growth rates, whereas for 42% the high-yield pathway is reduced at most by 10% (Figure 4B). Thus, at high growth rates FBAwMC predicts overflow-like metabolism in *E. coli*, whereas it predicts that the flux through the high-yield pathway is likely to be downregulated for *L. lactis *and *S. cerevisiae*.

### Acetate excretion makes E. coli use overflow-like switching

What could explain that *S. cerevisiae *and *L. lactis *downregulate high-yield metabolism at high growth rates, whereas *E. coli *uses overflow-like metabolism? Because we sampled from sets of prokaryotic crowding coefficients for *E. coli *and *L. lactis*, and from a eukaryotic dataset for *S. cerevisiae*, we first checked if the species-specific sets of crowding coefficients were responsible for our observations. We performed simulations with the *E. coli *network using the eukaryotic set of crowding coefficients and *vice versa*, and found that this did not affect our results. Therefore we conclude that the key difference between the three models is in the species-specific topology of the metabolic networks and their behavior in the presence of crowding, not in the specific values of crowding coefficients.

A key difference that sets *E. coli *apart from *L. lactis *and *S. cerevisiae *is shown in Figure 1. After converting glucose to pyruvate, *L. lactis *and *S. cerevisiae *either convert it into a waste product (ethanol) or further metabolize pyruvate to yield extra ATP. *L. lactis *converts two acetyl-coA into acetate and ethanol in parallel to retain redox balance, gaining (at most) one additional ATP per mole glucose. *S. cerevisiae *feeds pyruvate into the citric acid cycle and oxydative phosphorylation, gaining (at most) twenty-eight additional ATP and two GTP per mole glucose. Interestingly, *E. coli *has *three *choices: it can convert pyruvate into the waste product ethanol, it can metabolize pyruvate in the citric acid cycle, or it can gain one extra ATP in the conversion of acetyl-coA into acetate.

Although acetate production is a "cheap" way—in terms of the number of enzymes required—to produce additional ATP from pyruvate, it poses an additional challenge to *E. coli*. During formation of waste products (*i.e*., lactate or ethanol) the NADH produced in glycolysis or in the conversion from pyruvate to acetyl-coA is reduced back to NAD^+^. Thus such waste product formation is a "fast" way to restore a sufficiently high NAD^+^/NADH-ratio. Acetate production does not restore the NAD^+^/NADH-ratio, so acetate production might deplete the available NAD^+ ^in the cell. So, *E. coli *might keep oxidative phosphorylation running at fast growth rates (and consume oxygen) in order to profit from the extra ATP yield in acetate formation and restore the NAD^+^/NADH ratio.

**Figure 5:**
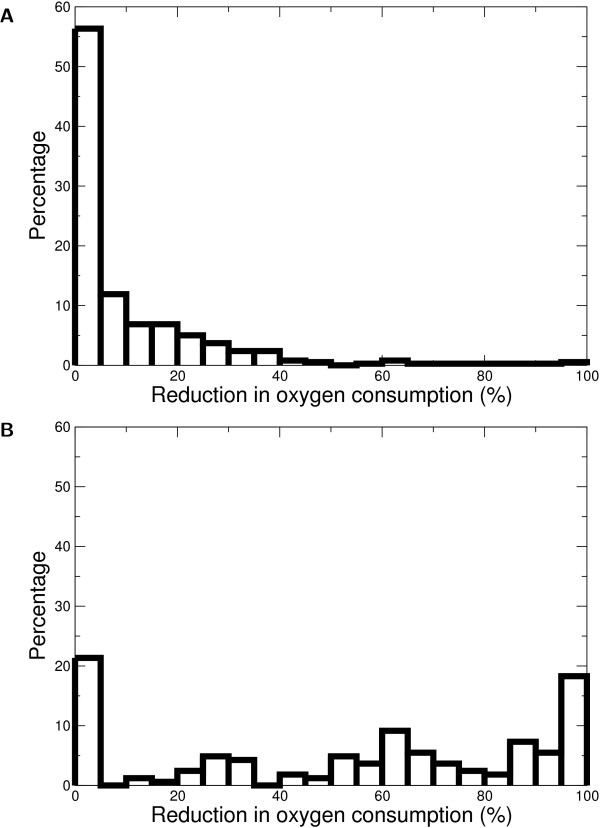
**Acetate fermentation *vs*. lactate and ethanol fermentation in *E. coli***. Histograms of the decrease in oxygen uptake rate, relative to the maximum oxygen uptake rate. A. Simulations that result in acetate fermentation (without lactate or ethanol fermentation); B. Simulations that result in ethanol or lactate fermentation.

This analysis suggests that, if *E. coli *ferments glucose into lactate or ethanol, its oxygen consumption will be reduced. We therefore studied the excretion patterns belonging to different sets of crowding coefficients more carefully. We found that, if a set of crowding coefficients results in acetate fermentation, without any ethanol or lactate fermentation, the cells continue to consume oxygen consumption during the switch. If a set of crowding coefficients results in lactate or ethanol fermentation, the consumption of oxygen is often reduced (Figure 5). Of the fraction of simulations of *E. coli *in which oxygen consumption is reduced during the metabolic switch (see Figure 4C) most produce ethanol and lactate, not acetate (data not shown). Thus, together these simulations are in agreement with the fact that *E. coli *needs to consume oxygen as an external electron acceptor during acetate fermentation in order to maintain a sufficiently high NAD^+^/NADH ratio [30]. If *E. coli *is genetically engineered to eliminate the TCA cycle and all NADH-reducing fermentation pathways (*i.e*., the strain cannot produce lactate or ethanol), it needs dissolved oxygen to grow [30].

**Figure 6:**
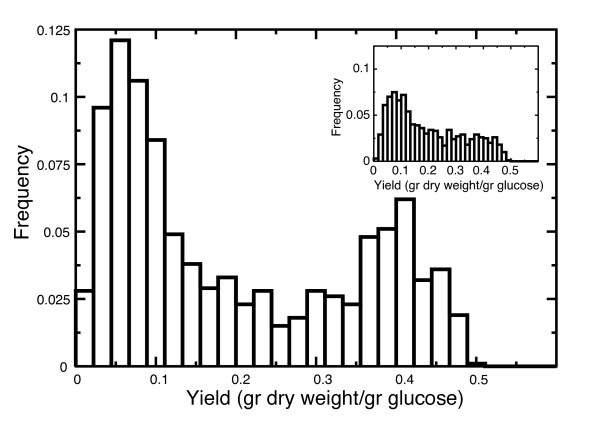
**Metabolic switching in model with blocked acetate excretion**. Distribution of growth yields predicted by the modified metabolic model with blocked acetate excretion of *E. coli *with 1000 randomly selected sets of crowding coefficients. Inset: growth yields calculated with original metabolic network of *E. coli*.

To further confirm the hypothesis that at high growth rates overflow metabolism is optimal in *E. coli *due to acetate excretion, we blocked acetate excretion in the FBAwMC model of *E. coli *(by setting the maximum efflux to zero) such that acetate production stalled, and recalculated the distribution of optimal growth yields. As Figure 6 demonstrates, in this simulation experiment the growth yields become bimodally distributed over the crowding coefficient samples, suggesting that *E. coli *can switch bimodally. Also, there are more sets of crowding coefficients that result in a decrease in flux through the high-yield pathway (Additional file 1: Figure S1). Apparently, after blocking the route for producing one extra ATP, it again becomes optimal to restore the NAD^+^/NADH ratio by producing the alternative waste products lactate or ethanol. This model observation agrees with experiments by De Mey *et al. *[31] who report increased lactate and ethanol excretion after reducing the carbon flow to acetate.

**Figure 7:**
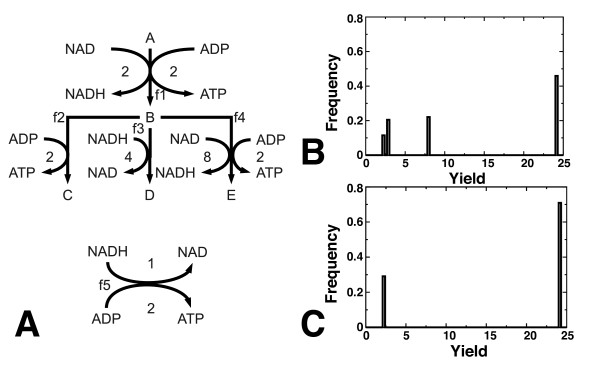
**Simplified network model of acetate production in *E. coli***. A. Simplified metabolic network. Reaction 1: glycolysis, reaction 2: acetate excretion, reaction 3: lactate/ethanol excretion, reaction 4: TCA-cycle, reaction 5: Oxidative phosphorylation; B. Growth yield distribution of the full simplified network; C. Growth yield distribution in simplified model with blocked acetate excretion.

To confirm that an additional ATP-producing pathway can indeed lead to an additional optimal growth mode, we developed a simplified metabolic network model [16], illustrated in Figure 7A. The simplified model has five reactions that represent glycolysis, lactate/ethanol excretion, acetate excretion, the TCA-cycle, and oxidative phosphorylation. Using FBAwMC we predicted the optimal yields for a sample of crowding coefficients. In this model, we found four metabolic modes (Figure 7B). After knocking out the acetate pathway, we found only two metabolic modes, a high-yield and a low-yield pathway (Figure 7C). Thus, also in this simplified model, acetate production introduces intermediate-yield metabolic modes.

## Discussion

We have computationally compared metabolic switching at high growth rates in *E. coli *with *L. lactis *and *S. cerevisiae. E. coli *shows overflow metabolism, meaning that at high growth rates it increases its metabolic rate by activating low-yield metabolic pathways in addition to the high-yield oxidative phosphorylation pathway. Instead, *L. lactis *and *S. cerevisiae *show metabolic switching: they suppress the flux through their high-yield pathways at high growth rates, relying mostly on low-yield metabolism. Our analysis suggests that a key difference between the two groups is the number of metabolic pathways yielding ATP, and the effect of these pathways on the NAD^+^/NADH-ratio. *L. lactis *and *S. cerevisiae *have two alternative pathways, an efficient, high-yield pathway—glycolysis followed by mixed acid fermentation or oxidative phosphorylation—and an inefficient, low-yield pathway—glycolysis followed by lactic acid or ethanol production. In both the low-yield and high-yield pathways, the NADH resulting from glycolysis is oxidized back to NAD^+^. In addition to lactate and ethanol fermentation and oxidative phosphorylation, *E. coli *has a second low-yield pathway: the conversion of pyruvate to acetate. This pathway yields one extra ATP over lactate fermentation, but it does not oxidize NADH, so the NAD^+^/NADH-ratio must be restored elsewhere. Our model suggests that in this case it is optimal to keep oxidative phosphorylation running, instead of calling in low-yield pathways to reduce NADH, *e.g*., lactate production.

To test the idea that acetate production is the cause of overflow metabolism in *E. coli*, we blocked acetate fermentation in the FBAwMC model. The distribution of predicted growth yields became more bimodal, and the proportion of cells that downregulated their high-yield pathway increased. Should we hence expect *E. coli *to downregulate its high-yield pathway at high growth rates, if its acetate production pathway were blocked experimentally? Note that FBAwMC predicts *optimal *growth rates. Thus it predicts the growth rates for organisms that have already evolved towards optimality. Our results would therefore suggest that mutated *E. coli *strain with blocked acetate fermentation would evolve downregulation of its high-yield pathway after selection for growth rate in cell culture experiments.

Our model results suggest that restoring the redox balance is key in metabolic switching, agrees with experimental observation. Vemuri *et al. *[32] overexpressed both NADH oxidase (NOX) and alternative oxidase (AOX) in *S. cerevisiae *and found that glycerol (for NOX) or ethanol formation (for AOX) were diminished. In another study, Vemuri *et al. *[33] increased oxidation of NADH by overexpressing NOX in *E. coli *and studied the effect on overflow metabolism. They found that overexpression of NOX strongly diminished acetate fermentation.

To check whether our model is consistent with these experiments, we mimicked them in the FBAwMC models for *E. coli *and *S. cerevisiae*. We introduced the reactions that NOX and AOX catalyze to the metabolic model and enforced a lower bound on their fluxes to mimic the effect of overexpression. We performed simulations of *S. cerevisiae *with ten thousand sets of crowding coefficients, of which only 110 crowding coefficient selections resulted in excretion of both ethanol and glycerol. We went on with these sets of crowding coefficients, because they best mimicked the wild-type phenotype that Vemuri *et al. *[33] used. The simulated overexpression of NOX or AOX reduced excretion of ethanol and glycerol in practically all of these 110 simulations (Table 1). Thus, in agreement with experiments, the FBAwMC model of *S. cerevisiae *suggests that excessive NADH breakdown reduces ethanol and glycerol fermentation. In the FBAwMC model of *E. coli *we found that increased oxidation of NADH could either result in a decreased or in an increased production of acetate, depending on the selection of crowding coefficients. Thus, the experiments of Vemuri *et al. *[33] do not corroborate nor falsify our model. The reason is that FBAwMC identifies optimal fluxes. In *E. coli*, during acetate fermentation some NADH is formed in the conversion of pyruvate to acetyl-coA. During NOX overexpression, the cells must boost NADH production to maintain optimal growth; this can be done either using the TCA-cycle or using acetate fermentation. Depending on the crowding coefficients, either way is optimal.

**Table 1:** Effect of NOX or AOX overexpression on low-yield metabolism

	NOX	AOX
*S. cerevisiae *ethanol	96%	98%
*S. cerevisiae *glycerol	100%	97%
*E. coli *acetate	16%	

We report the percentage of simulations that result in a decrease in acetate fermentation (for *E. coli*) or ethanol and glycerol fermentation (for *S. cerevisiae*)

The computational results presented in this paper are contingent on two underlying, biological assumptions of FBAwMC that may limit the applicability of our approach to strains growing in well-mixed, nutrient-rich lab conditions: a) evolution optimizes cells' growth rates instead of yields, and b) a solvent constraint (*i.e*., the number of enzymes "fitting" inside the cell) puts selective pressure on cells to evolve mechanisms to rapidly produce or remove enzymes for alternative metabolic pathways [22]. Thus FBAwMC implicitly assumes that evolution has shaped cells to make the optimal choice between alternative metabolic pathways.

The optimality assumption is not necessarily correct in all environments. Apart from the fact that evolution does not always lead to optimality [34], game theory suggests that spatial or seasonal environments favor maximization of growth yield [35,36]. Optimization of growth *rate*, as implicitly assumed in FBAwMC, is more likely applicable to homogeneous, non-seasonal environments, *i.e*., a chemostat [35,37]. Thus our simulations apply primarily to laboratory strains, which are adapted to well-mixed, nutrient-rich laboratory conditions. For natural strains, the maximization of growth yield that standard FBA assumes might be better applicable [20].

The second key assumption of FBAwMC, namely that cells have evolved regulation mechanisms to activate production of enzymatic machinery for the pathway giving optimal growth rate [22], relates closely to the explanation proposed by Molenaar *et al. *[12]. Using a minimal model of a self-replicator, they showed how a trade-off between the metabolic efficiency of a pathway, and the cost associated with producing the enzymes for that payway, can lead to a switch in metabolic strategy. Very recently, Zhuang *et al. *[38] proposed instead that competition for membrane space between glucose transporters and respiratory chain enzymes could be responsible for the metabolic switch between respirative and respiro-fermentative metabolism in *E. coli*. They introduced an alternative extension of FBA to accommodate for this effect. We anticipate that our results would hold if we used Zhuang *et al.*'s modification of FBA instead of FBAwMC. A requirement for our results is that the sum of a set of key metabolic fluxes is constrained. In FBAwMC this constraint is proposed to be due to the limited enzyme solvent capacity in the cytosol [12,17]. Mathematically, Zhuang *et al. *[38] propose a very similar constraint, but argue it is due to competition for membrane space between glucose enzymes and respiratory chain enzymes. In fact, the explanation proposed by Vazquez *et al. *[17] may be more generally applicable, because Zhuang *et al.*'s rationale would not hold if glucose transporters and respiratory chain enzymes do not share the same membranes as, *e.g*., in eukaryotes.

## Conclusions

Why, at high rates, do some microbes use low-yield metabolism in addition to the high-yield pathway—overflow metabolism—whereas other microbes downregulate their high-yield pathways? Here we show that maintaining redox balance is key to understanding overflow metabolism in *E. coli*. Microbes that use low-yield pathways converting NADH back to NAD, including *L. lactis *and *S. cerevisiae*, are expected to downregulate their high-yield pathways at high growth rates. *E. coli *can get one extra ATP using acetate secretion; doing so it must keep the oxidative phosphorylation pathway running to restore redox balance, giving rise to "overflow-like" metabolism.

## Methods

### Flux balance analysis with molecular crowding

We have used FBAwMC [17,22] to predict growth, uptake and excretion rates in *S. cerevisiae*, *E. coli *and *L. lactis*, using the genome-scale metabolic models published in [26,25] and [24], respectively. We downloaded the *E. coli *and *S. cerevisiae *model from the BiGG database [39]http://bigg.ucsd.edu/. The *L. lactis *model was downloaded from the Supplementary Materials in Oliveira *et al. *[24].

FBAwMC assumes that the metabolic network is in steady state

(2) $$\frac{d\vec{x}}{dt}=S\;.\;\vec{f}=0,$$

where $\vec{x}$ is a vector of all metabolites, $\vec{f}$is a vector describing the metabolic flux through each reaction in the network, and *S *the stoichiometric matrix. *S *is defined as follows: if reaction *i *produces *n *metabolites of type *j*, then *S_ij _*≡ *n *; *S_ij _*≡ - *n *for consumption of metabolite *j*; otherwise *S_ij _*≡ 0. FBAwMC attempts to find a solution F of Eq. 2 that maximizes an objective function, given a set of constraints. In this study, we always optimize for growth rate. We also incorporate constraints on the individual fluxes:

(3) $$f_{lb,n}\leq {\text{F}}_{n}\leq f_{ub,n},$$

where *f_lb, n _*is the minimal flux and *f_ub, n _*the maximal flux through reaction *n*. Furthermore, a constraint on the total flux through the network is added to account for the limited amount of enzymes in any given cell, given by

(4) $$\sum c_{n}f_{n}\leq V_{prot}.$$

Here $c_{n}\equiv \frac{Mv_{n}}{Vb_{n}}$is the "crowding coefficient", *M *the cell mass, *V *the cell volume, *v_n _*the molar volume of the enzyme catalysing reaction *n *and *b_n _*a parameter describing the proportionality between enzyme concentration and flux. For a derivation of Eq. 3 see Beg *et al. *[22]. *V_prot _*is a constant (0 ≤ *V_prot _*≤ 1) representing the volume fraction of macromolecules devoted to metabolic enzymes. We fit *V_prot _*to the experimentally observed growth rate and glucose uptake rate. Additional file 2: Table S1 lists the results of this fitting procedure. Interestingly, *V_prot _*values are very similar across different organisms, ranging from 0.15-0.2. Note that Vazquez *et al. *[17] assumed that *V_prot _*= 1, which we believe is unrealistic, because not only metabolic enzymes fill the cell's cytoplasm. Linear programming efficiently solves this problem, but the solution is not necessarily unique.

### Crowding coefficients

To obtain the crowding coefficients *c_i _*we adopted the approach of Vazquez *et al. *[17]. The molar volume *v_i _*can be estimated from the molar masses of the enzymes using a specific protein volume of 0.73 ml/g. *b_i _*depends on the concentration of metabolites and on the turnover numbers of the enzymes (for example in a Michaelis-Menten way, where $b=V_{max}\frac{S}{S+K_{M}},$here, we would estimate *b = V_max_*). Following Vazquez *et al. *[17], we constructed a distribution of crowding coefficients from turnover numbers and enzyme masses. We obtained turnover numbers from enzyme database Brenda [28], enzyme masses from MetaCyc [27]. The distribution of crowding coefficients we then obtained using the relationship $c_{i}\equiv \frac{Mv_{i}}{Vb_{i}}.$The turnover numbers and enzyme masses used are given in Additional file 3: Figure S2.

As there is insufficient data for *L. lactis *we used *E. coli *crowding coefficients for this organism as well. The turnover numbers, both for *E. coli *and *S. cerevisiae *varied over orders of magnitudes. Importantly, a few enzyme-substrate combinations had extremely low turnover numbers that effectively stopped the reactions. Because these turnover numbers typically occurred for non-metabolic reactions (*e.g*., DNA repair) or for non-typical substrates of metabolic enzymes, we only used turnover numbers of metabolic enzymes and for each enzyme we only kept the highest available turnover number and left out enzymes with turnover number smaller than 0.01/s. We used wild-type turnover numbers if reported. The resulting distribution of crowding coefficients for *E. coli *was similar to the distribution found by Vazquez *et al. *[17] (see Additional file 4: Figure S3). For *S. cerevisiae *we found a similar distribution as for *E. coli *(Additional file 5: Figure S4).

### In silico growth experiments

We initiated each simulation with randomly select crowding coefficients from the obtained distributions. We assigned a crowding coefficient of 0 to non-enzymatic reactions. The COBRA Toolbox [40] was used to perform FBAwMC in Matlab, with the GNU Linear Programming Kit as linear programming solver http://www.gnu.org/software/glpk.

The *in silico *growth media included the vitamins, nucleotides and minerals required for optimal growth. For the constraints on the reactions used in the simulations, we refer to Additional file 6: Table S2. Because *L. lactis *cannot synthesize many amino acids we must supply them in the *in silico *growth medium. In order to ensure that cells are not limited by amino acid uptake, we constrained the maximal amino acid uptake rates to the biomass content of that amino acid multiplied by twice the (experimentally observed) maximal growth rate. In this way, the maximal amino acid uptake rate suffices for twice the experimentally observed growth rate.

Matlab code to reproduce the simulations are included in Additional file 7. The COBRA Toolbox [40] and a linear programming solver are required.

## Abbreviations

AOX: Alternative oxidase; ATP: Adenosine triphosphate; BiGG: Biochemical Genetic and Genomic knowledgebase of large scale metabolic reconstructions; CO_2_: Carbon dioxide; COBRA: COnstraints Based Reconstruction and Analysis; FBA: Flux-balance analysis; FBAwMC: Flux-balance analysis with molecular crowding; GTP: Guanosine-5'-triphosphate; NAD^+^: Nicotinamide Adenine Dinucleotide; NADH: Reduced form of NAD^+^; NOX: NADH oxidase.

## Competing interests

The authors declare that they have no competing interests.

## Authors' contributions

MvH designed the model and performed the simulations. RM and MvH conceived of the study and drafted the manuscript. All authors read and approved the final manuscript.

## Supplementary Material

Additional file 1 **Figure S1**. Histogram of decrease in oxygen uptake for *E. coli*, when acetate excretion is allowed (black) and knocked out (red). When acetate excretion is knocked out, there are more simulations that become fully high-yield, but also more that stop consuming oxygen. Click here for file

Additional file 2 **Table S1**. Table describing the summary of fitting *V_prot _*to experimental growth rate and glucose uptake rate. For every organism, we varied *V_prot _*(volume fraction of macromolecules devoted to metabolic enzymes) between 0 and 1 and performed, for each value of *V_prot_*, 1000 simulations with random sets of crowding coefficients. For the simulations described in this paper, we used the value of *V_prot _*that minimized ((*μ_max, fit _*- *μ_max, obs_*)/*μ_max, obs_*)^2^+((*Gup_max, fit _*- *Gup_max, obs_*)/*Gup_max, obs_*)^2^. Here, *μ_max, fit_*, *μ_max, obs _*are the fitted and observed maximal growth rate and *Gup_max, fit_*, *Gup_max, obs _*are the fitter and observed maximal glucose uptake rate. In this table, *P_ineff _*indicates the fraction of the 1000 simulations that exhibits low-yield metabolism, which was defined as having a growth yield < 0.3 gr/gr glucose. Experimental data is from Hoek *et al. *[29]; Thomas *et al. *[4]; Varma and Palsson [2]. Click here for file

Additional file 3 **Figure S2**. Excel file with turnover numbers and enzyme masses used to calculate the crowding coefficients. Click here for file

Additional file 4 **Figure S3**. Histograms of turnover numbers (1/s) (A) and crowding coefficients (gram DW hr/mmol) (B) of *E. coli*. A. All turnover numbers of *E. coli *in BRENDA (Chang *et al. *[28]); B. Crowding coefficients resulting from all turnover numbers of *E. coli *in BRENDA (Chang *et al. *[28]); C. Turnover numbers of *E. coli *used for the simulations; D. Crowding coefficients of *E. coli *used in the simulations; E. Turnover numbers as used in Vazquez *et al. *[17]; F. Crowding coefficients as used in Vazquez *et al. *[17]. Click here for file

Additional file 5 **Figure S4**. Histograms of turnover numbers (1/s) (A,C) and crowding coefficients (gram DW hr/mmol) (B,D) of *S. cerevisiae*. A. All turnover numbers of *S. cerevisiae *in BRENDA (Chang *et al. *[28]); B. Crowding coefficients resulting from all turnover numbers of *S. cerevisiae *in BRENDA (Chang *et al. *[28]); C. Turnover numbers of *S. cerevisiae *used for the simulations; D. Crowding coefficients of *S. cerevisiae *used in the simulations. Click here for file

Additional file 6 **Table S2**. Excel file describing, for every reaction, the lower and upper bounds used in the simulations. Click here for file

Additional file 7 **Mini-website with Matlab code and instructions for reproducing the simulations**. Click here for file

## References

[B1] van Dijken JP Weusthuis RA Pronk JT Kinetics of growth and sugar consumption in yeasts Antonie Van Leeuwenhoek 1993 63 3-4 343 352 10.1007/BF00871229 8279829

[B2] Varma A Palsson BØ Stoichiometric flux balance models quantitatively predict growth and metabolic by-product secretion in wild-type Escherichia coli W3110 Appl Environ Microbiol 1994 60 10 3724 3731 7986045 10.1128/aem.60.10.3724-3731.1994 PMC201879

[B3] Dauner M Storni T Sauer U Bacillus subtilis metabolism and energetics in carbon-limited and excess-carbon chemostat culture J Bacteriol 2001 183 24 7308 7317 10.1128/JB.183.24.7308-7317.2001 11717290 PMC95580

[B4] Thomas TD Ellwood DC Longyear VM Change from homo- to heterolactic fermentation by Streptococcus lactis resulting from glucose limitation in anaerobic chemostat cultures J Bacteriol 1979 138 109 117 108249 10.1128/jb.138.1.109-117.1979 PMC218245

[B5] Teusink B Wiersma A Molenaar D Francke C de Vos WM Siezen RJ Smid EJ Analysis of growth of Lactobacillus plantarum WCFS1 on a complex medium using a genome-scale metabolic model J Biol Chem 2006 281 52 40041 40048 10.1074/jbc.M606263200 17062565

[B6] Kim JW Dang CV Cancer's molecular sweet tooth and the Warburg effect Cancer Res 2006 66 18 8927 8930 10.1158/0008-5472.CAN-06-1501 16982728

[B7] Robergs RA Ghiasvand F Parker D Biochemistry of exercise-induced metabolic acidosis Am J Physiol Regul Integr Comp Physiol 2004 287 3 R502 R516 10.1152/ajpregu.00114.2004 15308499

[B8] Westerhoff HV Hellingwerf KJ Dam KV Thermodynamic efficiency of microbial growth is low but optimal for maximal growth rate P Natl Acad Sci USA 1983 80 305 309 10.1073/pnas.80.1.305 PMC393362 6572006

[B9] Tempest DW Neijssel OM Physiological and energetic aspects of bacterial metabolite overproduction FEMS Microbiol Lett 1992 79 1-3 169 176 1478453 10.1111/j.1574-6968.1992.tb14036.x

[B10] Russell JB Cook GM Energetics of bacterial growth: balance of anabolic and catabolic reactions Microbiol Rev 1995 59 48 62 7708012 10.1128/mr.59.1.48-62.1995 PMC239354

[B11] Russell JB The energy spilling reactions of bacteria and other organisms J Mol Microbiol Biotechnol 2007 13 1-3 1 11 10.1159/000103591 17693707

[B12] Molenaar D van Berlo R de Ridder D Teusink B Shifts in growth strategies reflect tradeoffs in cellular economics Mol Syst Biol 2009 5 323 19888218 10.1038/msb.2009.82 PMC2795476

[B13] Andersen KB Von Meyenburg K Are growth rates of Escherichia coli in batch cultures limited by respiration? J Bacteriol 1980 144 114 123 6998942 10.1128/jb.144.1.114-123.1980 PMC294601

[B14] Holms H Flux analysis and control of the central metabolic pathways in Escherichia coli FEMS Microbiol Rev 1996 19 2 85 116 10.1111/j.1574-6976.1996.tb00255.x 8988566

[B15] Heinrich R Montero F Klipp E Waddell TG Melendez-Hevia E Theoretical approaches to the evolutionary optimization of glycolysis: thermodynamic and kinetic constraints Eur J Biochem 1997 243 1-2 191 201 10.1111/j.1432-1033.1997.0191a.x 9030739

[B16] Schuster S Pfeiffer T Fell DA Is maximization of molar yield in metabolic networks favoured by evolution? J Theor Biol 2008 252 3 497 504 10.1016/j.jtbi.2007.12.008 18249414

[B17] Vazquez A Beg QK Demenezes MA Ernst J Bar-Joseph Z Barabasi AL Boros LG Oltvai ZN Impact of the solvent capacity constraint on E. coli metabolism BMC Syst Biol 2008 2 7 10.1186/1752-0509-2-7 18215292 PMC2270259

[B18] Vazquez A Liu J Zhou Y Oltvai ZN Catabolic efficiency of aerobic glycolysis: the Warburg effect revisited BMC Syst Biol 2010 4 58 10.1186/1752-0509-4-58 20459610 PMC2880972

[B19] Merico A Sulo P Piskur J Compagno C Fermentative lifestyle in yeasts belonging to the Saccharomyces complex FEBS J 2007 274 4 976 989 10.1111/j.1742-4658.2007.05645.x 17239085

[B20] Fuhrer T Fischer E Sauer U Experimental identification and quantification of glucose metabolism in seven bacterial species J Bacteriol 2005 187 5 1581 1590 10.1128/JB.187.5.1581-1590.2005 15716428 PMC1064017

[B21] Edwards JS Palsson BØ The Escherichia coli MG1655 in silico metabolic genotype: its definition, characteristics, and capabilities P Natl Acad Sci USA 2000 97 10 5528 5533 10.1073/pnas.97.10.5528 PMC25862 10805808

[B22] Beg QK Vazquez A Ernst J de Menezes MA Bar-Joseph Z Barabasi AL Oltvai ZN Intracellular crowding defines the mode and sequence of substrate uptake by Escherichia coli and constrains its metabolic activity P Natl Acad Sci USA 2007 104 31 12663 12668 10.1073/pnas.0609845104 PMC1937523 17652176

[B23] Shlomi T Benyamini T Gottlieb E Sharan R Ruppin E Genome-scale metabolic modeling elucidates the role of proliferative adaptation in causing the Warburg effect PLoS Comput Biol 2011 7 3 e1002018 10.1371/journal.pcbi.1002018 21423717 PMC3053319

[B24] Oliveira AP Nielsen J Forster J Modeling Lactococcus lactis using a genome-scale flux model BMC Microbiol 2005 5 39 10.1186/1471-2180-5-39 15982422 PMC1185544

[B25] Feist AM Henry CS Reed JL Krummenacker M Joyce AR Karp PD Broadbelt LJ Hatzimanikatis V Palsson BØ A genome-scale metabolic reconstruction for Escherichia coli K-12 MG1655 that accounts for 1260 ORFs and thermodynamic information Mol Syst Biol 2007 3 121 17593909 10.1038/msb4100155 PMC1911197

[B26] Duarte NC Herrgard MJ Palsson BØ Reconstruction and validation of Saccharomyces cerevisiae iND750, a fully compartmentalized genome-scale metabolic model Genome Res 2004 14 7 1298 1309 10.1101/gr.2250904 15197165 PMC442145

[B27] Caspi R Foerster H Fulcher CA Kaipa P Krummenacker M Latendresse M Paley S Rhee SY Shearer AG Tissier C Walk TC Zhang P Karp PD The MetaCyc Database of metabolic pathways and enzymes and the BioCyc collection of Pathway/Genome Databases Nucleic Acids Res 2008 36 Database D623 D631 17965431 10.1093/nar/gkm900 PMC2238876

[B28] Chang A Scheer M Grote A Schomburg I Schomburg D BRENDA, AMENDA and FRENDA the enzyme information system: new content and tools in 2009 Nucleic Acids Res 2009 37 (Database issue):D588 D592 10.1093/nar/gkn820 PMC2686525 18984617

[B29] Hoek PV Dijken JPV Pronk JT Effect of specific growth rate on fermentative capacity of baker's yeast Appl Environ Microbiol 1998 64 11 4226 4233 9797269 10.1128/aem.64.11.4226-4233.1998 PMC106631

[B30] Causey TB Zhou S Shanmugam KT Ingram LO Engineering the metabolism of Escherichia coli W3110 for the conversion of sugar to redox-neutral and oxidized products: homoacetate production P Natl Acad Sci USA 2003 100 3 825 832 10.1073/pnas.0337684100 PMC298686 12556564

[B31] Mey MD Lequeux GJ Beauprez JJ Maertens J Horen EV Soetaert WK Vanrolleghem PA Vandamme EJ Comparison of different strategies to reduce acetate formation in Escherichia coli Biotechnol Progr 2007 23 5 1053 1063 10.1021/bp070170g 17715942

[B32] Vemuri GN Eiteman MA McEwen JE Olsson L Nielsen J Increasing NADH oxidation reduces overflow metabolism in Saccharomyces cerevisiae P Natl Acad Sci USA 2007 104 7 2402 2407 10.1073/pnas.0607469104 PMC1892921 17287356

[B33] Vemuri GN Altman E Sangurdekar DP Khodursky AB Eiteman MA Overflow metabolism in Escherichia coli during steady-state growth: transcriptional regulation and effect of the redox ratio Appl Environ Microbiol 2006 72 5 3653 3661 10.1128/AEM.72.5.3653-3661.2006 16672514 PMC1472329

[B34] Bull JJ Wang IN Optimality models in the age of experimental evolution and genomics J Evolution Biol 2010 23 9 1820 1838 10.1111/j.1420-9101.2010.02054.x PMC3004014 20646132

[B35] Pfeiffer T Schuster S Bonhoeffer S Cooperation and competition in the evolution of ATP-producing pathways Science 2001 292 5516 504 507 10.1126/science.1058079 11283355

[B36] MacLean RC Gudelj I Resource competition and social conflict in experimental populations of yeast Nature 2006 441 7092 498 501 10.1038/nature04624 16724064

[B37] Pfeiffer T Schuster S Game-theoretical approaches to studying the evolution of biochemical systems Trends Biochem Sci 2005 30 20 25 10.1016/j.tibs.2004.11.006 15653322

[B38] Zhuang K Vemuri GN Mahadevan R Economics of membrane occupancy and respiro-fermentation Mol Syst Biol 2011 7 500 21694717 10.1038/msb.2011.34 PMC3159977

[B39] Schellenberger J Park JO Conrad TM Palsson BØ BiGG: a Biochemical Genetic and Genomic knowledgebase of large scale metabolic reconstructions BMC Bioinformatics 2010 11 213 10.1186/1471-2105-11-213 20426874 PMC2874806

[B40] Becker SA Feist AM Mo ML Hannum G Palsson BØ Herrgard MJ Quantitative prediction of cellular metabolism with constraint-based models: the COBRA Toolbox Nat Protoc 2007 2 3 727 738 10.1038/nprot.2007.99 17406635

