# Supplementary figures and images for: Redox balance is key to explaining full vs. partial switching to low-yield metabolism
Source: BMC Syst Biol. 2012 Mar 24;6:22. doi: 10.1186/1752-0509-6-22 (PMC3384451; doi:10.1186/1752-0509-6-22)

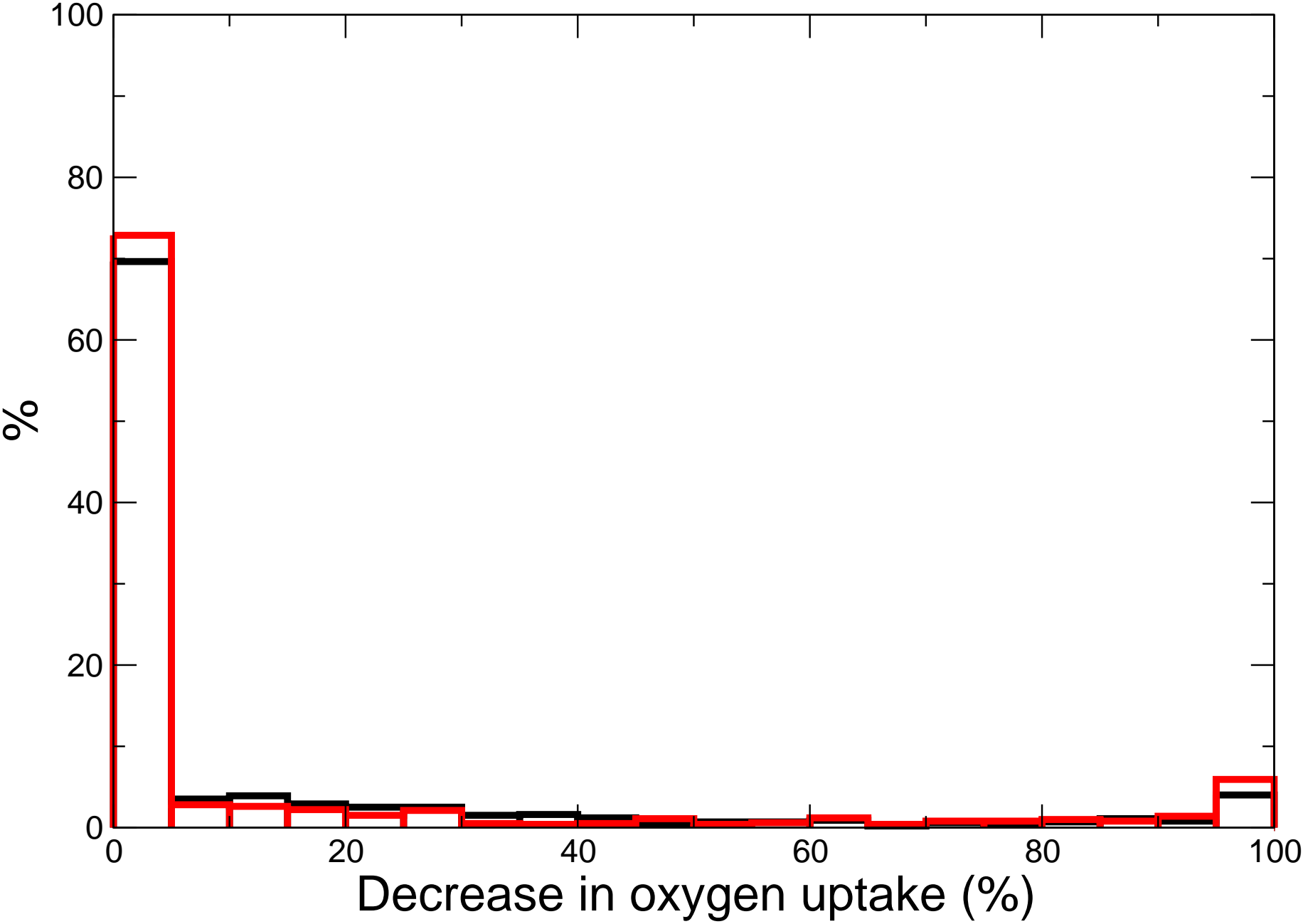

Supplement: Additional file 1 — Figure S1. Histogram of decrease in oxygen uptake for E. coli, when acetate excretion is allowed (black) and knocked out (red). When acetate excretion is knocked out, there are more simulations that become fully high-yield, but also more that stop consuming oxygen. [file 1752-0509-6-22-S1.PDF]

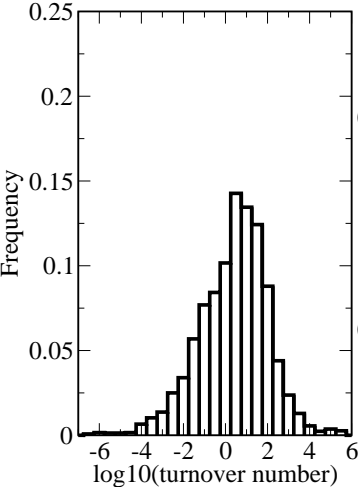

*A*

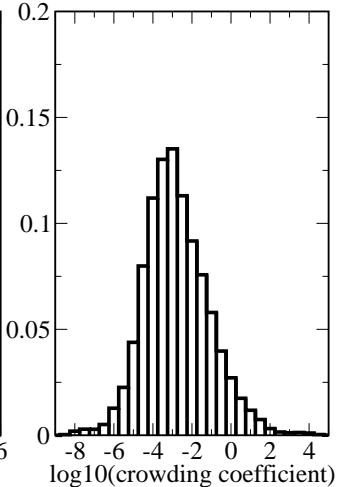

*B*

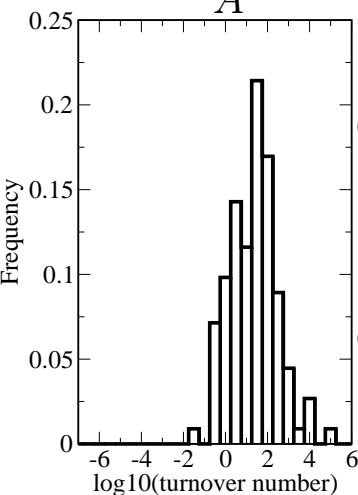

*C*

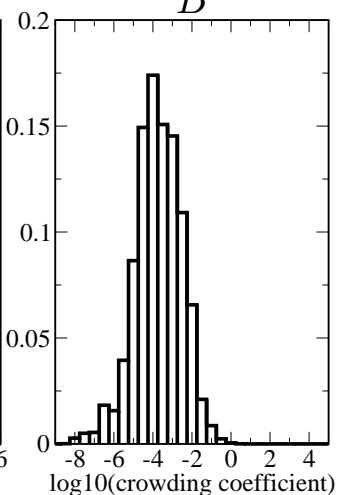

*D*

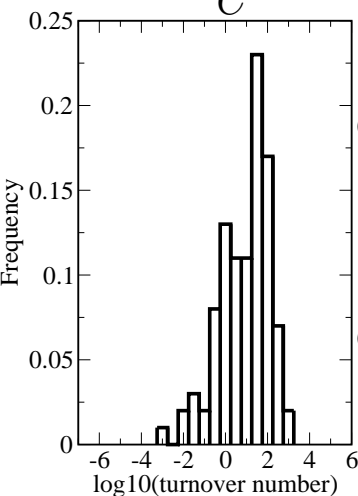

*E*

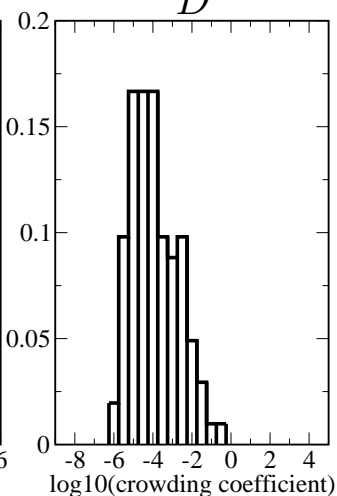

*F*

Supplement: Additional file 4 — Figure S3. Histograms of turnover numbers (1/s) (A) and crowding coefficients (gram DW hr/mmol) (B) of E. coli. A. All turnover numbers of E. coli in BRENDA (Chang et al. [28]); B. Crowding coefficients resulting from all turnover numbers of E. coli in BRENDA (Chang et al. [28]); C. Turnover numbers of E. coli used for the simulations; D. Crowding coefficients of E. coli used in the simulations; E. Turnover numbers as used in Vazquez et al. [17]; F. Crowding coefficients as used in Vazquez et al. [17]. [file 1752-0509-6-22-S4.PDF]

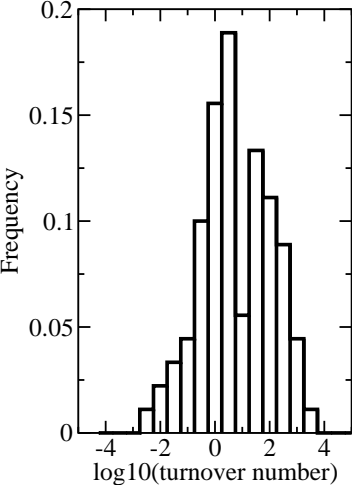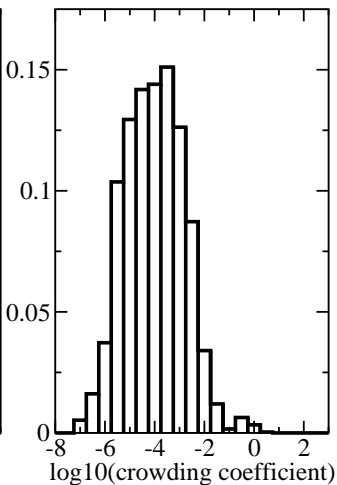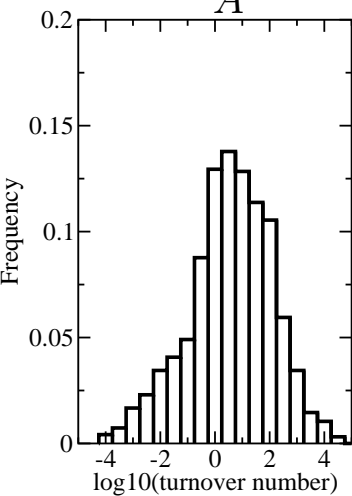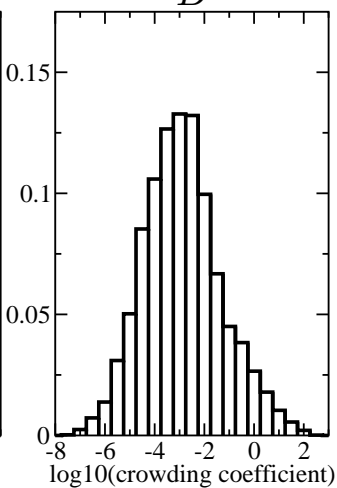

Supplement: Additional file 5 — Figure S4. Histograms of turnover numbers (1/s) (A,C) and crowding coefficients (gram DW hr/mmol) (B,D) of S. cerevisiae. A. All turnover numbers of S. cerevisiae in BRENDA (Chang et al. [28]); B. Crowding coefficients resulting from all turnover numbers of S. cerevisiae in BRENDA (Chang et al. [28]); C. Turnover numbers of S. cerevisiae used for the simulations; D. Crowding coefficients of S. cerevisiae used in the simulations. [file 1752-0509-6-22-S5.PDF]
