# Supplementary material for: Redox balance is key to explaining full vs. partial switching to low-yield metabolism
Source: BMC Syst Biol. 2012 Mar 24;6:22. doi: 10.1186/1752-0509-6-22 (PMC3384451; doi:10.1186/1752-0509-6-22)
Supplement: Additional file 2 — Table S1. Table describing the summary of fitting Vprot to experimental growth rate and glucose uptake rate. For every organism, we varied Vprot (volume fraction of macromolecules devoted to metabolic enzymes) between 0 and 1 and performed, for each value of Vprot, 1000 simulations with random sets of crowding coefficients. For the simulations described in this paper, we used the value of Vprot that minimized ((μmax, fit - μmax, obs)/μmax, obs)2+((Gupmax, fit - Gupmax, obs)/Gupmax, obs)2. Here, μmax, fit, μmax, obs are the fitted and observed maximal growth rate and Gupmax, fit, Gupmax, obs are the fitter and observed maximal glucose uptake rate. In this table, Pineff indicates the fraction of the 1000 simulations that exhibits low-yield metabolism, which was defined as having a growth yield < 0.3 gr/gr glucose. Experimental data is from Hoek et al. [29]; Thomas et al. [4]; Varma and Palsson [2]. [file 1752-0509-6-22-S2.PDF]

organism	$\mu_{max,fit}$ h^{-1}	$\mu_{max,obs}$ h^{-1}	$Gup_{max,fit}$ $\frac{mmol}{gDWh}$	$Gup_{max,obs}$ $\frac{mmol}{gDWh}$	V_{Prot}	P_{ineff}
S. cerevisiae	0.41	0.40	9.2	11.1	0.15	32%
L. lactis	0.89	0.76	19.0	24.6	0.2	44%
E. coli	0.32	0.69	15.1	11.0	0.2	74%
