# Supplementary material for: Redox balance is key to explaining full vs. partial switching to low-yield metabolism
Source: BMC Syst Biol. 2012 Mar 24;6:22. doi: 10.1186/1752-0509-6-22 (PMC3384451; doi:10.1186/1752-0509-6-22)
Supplement: Additional file 7 — Mini-website with Matlab code and instructions for reproducing the simulations. [file 1752-0509-6-22-S7.ZIP › AdditionalFile7/index.html]

USAGE.html
