# Supplementary material for: Redox balance is key to explaining full vs. partial switching to low-yield metabolism
Source: BMC Syst Biol. 2012 Mar 24;6:22. doi: 10.1186/1752-0509-6-22 (PMC3384451; doi:10.1186/1752-0509-6-22)
Supplement: Additional file 7 — Mini-website with Matlab code and instructions for reproducing the simulations. [file 1752-0509-6-22-S7.ZIP › AdditionalFile7/USAGE.html]

Home.html


## Matlab code for Van Hoek and Merks: Redox balance is key to explaining full *vs.* partial switching to low-yield metabolism; *BMC Systems Biology*

Use the Matlab code in this Additional File to reproduce the
simulations shown in our publication.

### Usage

Install the COBRA Toolbox (Becker et al., Nature Protocols 2007 2(3):727-38).
The COBRA Toolbox can be downloaded from http://opencobra.sourceforge.net/openCOBRA/Welcome.html.   
Follow the installation instructions on their website.   
Install the GNU Linear Programming Kit (GLPK, http://www.gnu.org/software/glpk/).  
Add the COBRA Toolbox directories and the GLPK directories to your MATLAB path (see the file startupnew.m).
  

### Matlab files

- Figure 2A and Figure 4A: llactis\_switch.m- Figure 2B and Figure 4B: scerevisae\_switch.m- Figure 2C and Figure 4C: ecoli\_switch.m- Figure 3A: llactis\_yields.m- Figure 3B: scerevisiae\_yields.m- Figure 3C: ecoli\_yields.m- Figure 5: ecoli\_yields\_noacetate.m- Figure 6: ecoli\_acetate\_ethanollactate.m- Figure 7: solve\_toymodel.m- Table 1: ecoli\_nox.m, scerevisiae\_nox\_aox.m- Additional File 1: ecoli\_oxdown.m

### Metabolic networks

- *Lactococcus lactis*: llactis\_input.mat- *Saccharomyces cerevisiae*: scerevisiae\_input.mat- *Escherichia coli*: ecoli\_input.mat- Simplified model of *E. coli*: toymodel\_input.mat
